# PHLPP2: A Prognostic Biomarker in Adenocarcinoma of the Rectum

**DOI:** 10.5152/tjg.2023.23189

**Published:** 2023-10-01

**Authors:** Keju Xie

**Affiliations:** Department of Plastic Surgery, The Affiliated Hospital of Shaoxing University, Shaoxing Municipal Hospital, Shaoxing, China

**Keywords:** Adenocarcinoma of the rectum, PH domain and leucine-rich repeat protein phosphatase 2, PI3K/AKT signaling, autophagy, survival ratio, colorectal cancer

## Abstract

**Background/Aims:**

Adenocarcinoma of the rectum (READ) is typically diagnosed at advanced stages due to a lack of early-onset specific features.

**Materials and Methods:**

The study used bioinformatics analysis of READ ribonucleic acid sequencing data from The Cancer Genome Atlas database to identify differentially expressed genes (DEGs). Overlapping genes between DEGs and autophagy-associated genes were screened for prognosis-associated DEGs, which were then validated in the OncoLnc database.

**Results:**

A total of 129 autophagy-associated DEGs were identified, with 17 genes found to be associated with READ prognosis. Multivariate Cox regression analysis revealed that only the PHLPP2 gene was significantly associated with READ prognosis (hazard ratio = 0.442, *P* = .026), and its low expression correlated with low survival in patients with brain lower-grade glioma (*P* = .00623) and pancreatic adenocarcinoma (*P* = .00109).

**Conclusions:**

PHLPP2 expression may serve as a READ-specific prognostic biomarker and is involved in the PI3K-Akt signaling pathway.

**Main Points**

- Expression of PHLPP2 was downregulated in tissues of rectal adenocarcinoma (READ).
- Low PHLPP2 expression was correlated with a lower survival ratio in patients with READ.
- PHLPP2 interacted with the downregulated AKT3 in READ samples.

## Introduction

Adenocarcinoma of the rectum (READ) is a type of colorectal cancer (CRC) that originates in the rectum and rectal tube. It is one of the malignant tumors with poor prognosis in patients with advanced tumors. The 5-year survival ratio of patients diagnosed with early READ was 70%-90%, and that of patients with advanced READ was less than 60%,^1^ even less than 30%.^2,3^ However, most READs were diagnosed at advanced stages due to the lack of specific features of early READ.

Over the past 2 decades, a number of high-quality studies have shown that clinical factors are associated with the early-onset, progression, and prognosis of CRC,^4-6^ while little data has been reported on READ.^2,7,8^ Also, the analysis of genetic research is also unfair. For instance, various genetic factors including messenger ribonucleic acids (mRNAs), microRNAs (miRNAs), long noncoding RNAs, and mutations have shown an association with the prognosis of CRC.^9-14^ However, the number of reports focusing on the features, treatment responses, and prognosis of READ is relatively small.^15-18^ Therefore, identification of diagnostic or prognostic biomarkers for READ is still necessary.

Advances in computational bioinformatics have attracted a great amount of interest in the field of cancer research. Computational bioinformatics analysis is of great value in identifying potent prognostic biomarkers, and some are likely to be used clinically. For instance, Pan et al^19^ showed that integrating 5 CRC-related miRNAs (including miR-15b, miR-17, miR-21, miR-26b, and miR-145) and serum carcinoembryonic antigen provided good diagnostic performance in CRC prognosis. Hansen et al^20^ showed that patients with a loss mutation of caudal-related homoeobox transcription factor 2 (CDX2) had a poor prognosis in 2 Denmark clinical cohorts. The correlation of CDX2 loss mutation with colon cancer prognosis had been previously reported by Dalerba et al^21^ using bioinformatics analysis. Since computational bioinformatics facilitates the identification of potential biomarkers, its popularization will provide a valuable reference to the features of cancers with unknown or unclear pathogenesis, including READ.

This study aimed to identify potential prognostic biomarkers in READ based on computational bioinformatics analysis, which would provide a novel genetic reference to the pathogenesis and development of READ.

## Materials and Methods

### The Cancer Genome Atlas Data Collection

The RNA-seq data (Illumina HiSeq 2000 RNA Sequencing) were extracted from The Cancer Genome Atlas (TCGA) database. A total of 177 samples, including 158 samples with clinical information and 9 nontumor adjacent tissues, were analyzed. The data files were downloaded and used for further analyses.

### Identification of Differentially Expressed Genes

The differentially expressed genes (DEGs) in the READ tumor samples were identified using the R Limma package (https://bioconductor.org/packages/release/bioc/html/limma.html; version 3.6.1). Differentially expressed genes were screened out according to the following criteria: log_2_(fold change) > 1, *P* < .05, and false discovery rate (FDR) < .05.

### Extraction of Autophagy-Associated Genes

To understand the molecular changes mediated by autophagy, the autophagy-associated genes were extracted from the Comparative Toxicogenomics Database (CTD; http://ctdbase.org/about/; 2020 update) using the search keyword “autophagy” and the National Center for Biotechnology Information (NCBI) gene database with the following search phrase: (autophagy) AND “Homo sapiens.” Also, the items included in the Human Autophagy Database (HADb; http://www.autophagy.lu/) were downloaded. Then, the genes overlapped between DEGs and at least 1 of the 3 databases (CTD, HADb, and NCBI) were retained and used for further analyses.

### Construction of the Protein–Protein Interaction Network

The protein–protein interaction (PPI) network was constructed for the autophagy-associated DEGs to show the potential interactions between the genes. The predictive interaction pairs were extracted from the STRING source (https://string-db.org/cgi/input.pl; version 11.0). Interaction pairs with a score of higher than 0.4 were downloaded. Then, the PPI network was constructed using the Cytoscape (http://apps.cytoscape.org/apps/all; version 3.8.0). The significant modules with a module score of higher than 5.0 in the PPI network were identified using the MCODE plugin in the Cytoscape (http://apps.cytoscape.org/search?q=MCODE).

### Identification of Adenocarcinoma of the Rectum Prognosis-Associated Genes

The prognosis-associated genes in READ were identified using the Cox regression analysis based on the TCGA cohort. Briefly, the expression profiles of the autophagy-associated DEGs including in the PPI network, clinical overall survival time, and death data were extracted from the TCGA cohort. Then, the univariate and multivariate Cox regression analyses were used to screen the prognosis-associated DEGs. Significant items were identified when *P* < .05. The associations of the above selected prognosis-associated DEGs with the overall survival and prognosis in other cancers were validated in the OncoLnc database (http://www.oncolnc.org/).

### Searching of the Pathways Related to Prognosis-Associated Differentially Expressed Genes

At last, we constructed the molecular regulatory network involving the prognosis-associated DEGs based on the searching result in the KEGG PATHWAY Database (https://www.kegg.jp/kegg/pathway.html). The pathways associated with the prognosis-associated DEGs and the nodes that interacted with them in the PPI were extracted from the KEGG database. Then, the mRNA-pathway regulatory network was constructed using the Cytoscape (version 3.8.0).

### Statistical Analysis

The difference in the expression level of DEGs between tumors with different clinical stages and metastatic statuses was analyzed using the nonparameter Mann–Whitney *U*-test or the Kruskal–Wallis H test. Also, the Cox regression analysis was performed using the Statistical Package for the Social Sciences 22.0 software (IBM corp., Armonk, NY, USA). Hazard ratio and 95% CI values were analyzed. For all analyses, a significant difference was defined at *P* < .05.

## Results

### Screening of the Differentially Expressed Genes in Adenocarcinoma of the Rectum Tumor

**Figure 1.**
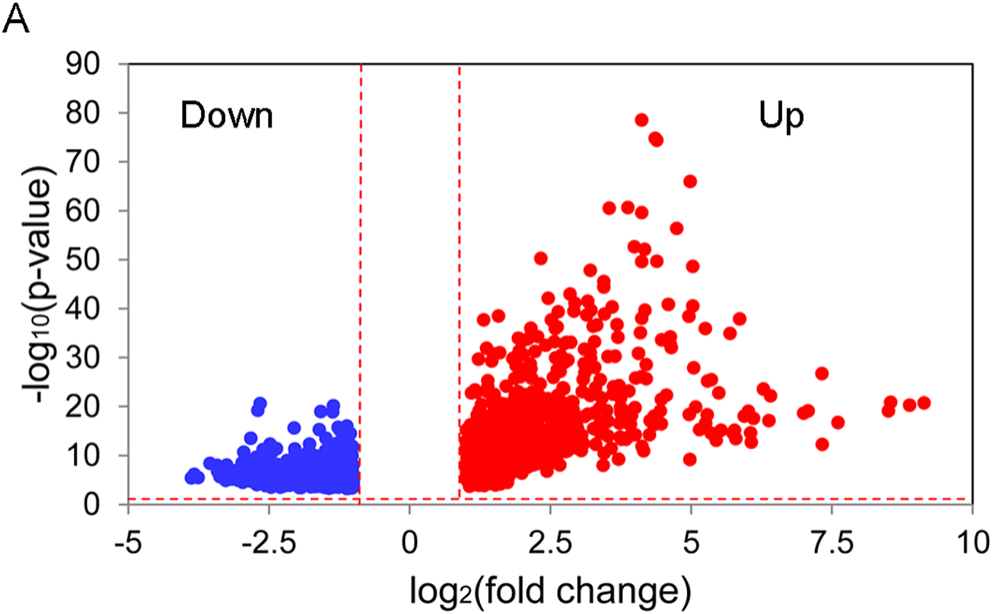
The volcano figure of the DEGs in the TCGA tumor samples compared with the controls. Upregulated (log_2_[fold change] >1, *P* < .05, and false discovery rate (FDR) < .05) and downregulated DEGs (log_2_[fold change] <−1, *P* < .05, and FDR < .05) were indicated as red and blue nodes, respectively. DEGs, differentially expressed genes; TCGA, The Cancer Genome Atlas.

Using the aforementioned criteria, a total of 1790 DEGs in tumor samples were screened out from the TCGA cohort. The volcano figure of the DEGs is shown in Figure 1.

### Identification of Autophagy-Associated Differentially Expressed Genes in Adenocarcinoma of the Rectum Tumor

**Figure 2.**
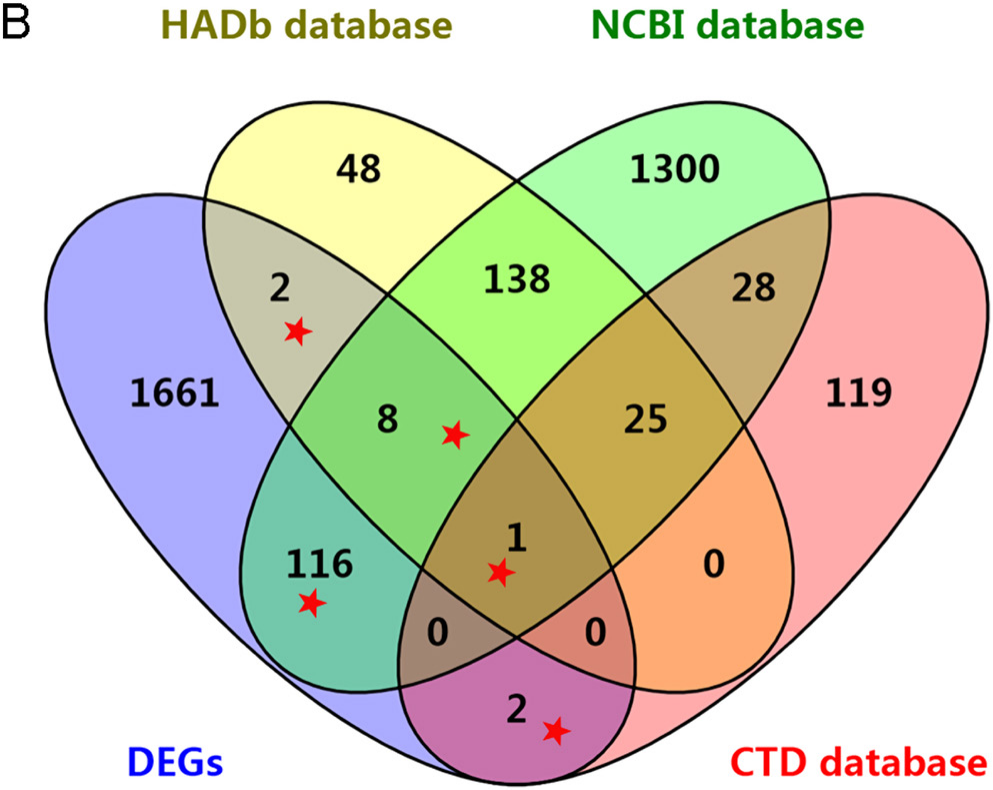
The Venn diagram representing the DEGs that overlapped between the autophagy-associated genes in the 3 databases. Overlapping genes indicated by red stars (n = 129) were the identified autophagy-associated DEGs and used for further analyses. CTD, Comparative Toxicogenomics Database; DEGs, differentially expressed genes; HADb, Human Autophagy Database; NCBI, National Center for Biotechnology Information.

Based on the searching in CTD and NCBI databases, 175 and 1619 autophagy genes were identified. Also, 232 autophagy-associated genes were downloaded from the HADb (Figure 2). The Venn diagram indicated that 129 DEGs were included in at least 1 of the 3 databases. The list of the 129 autophagy-associated DEGs is shown in Supplementary Table 1.

### Protein–Protein Interaction Construction and Module Identification

**Figure 3.**
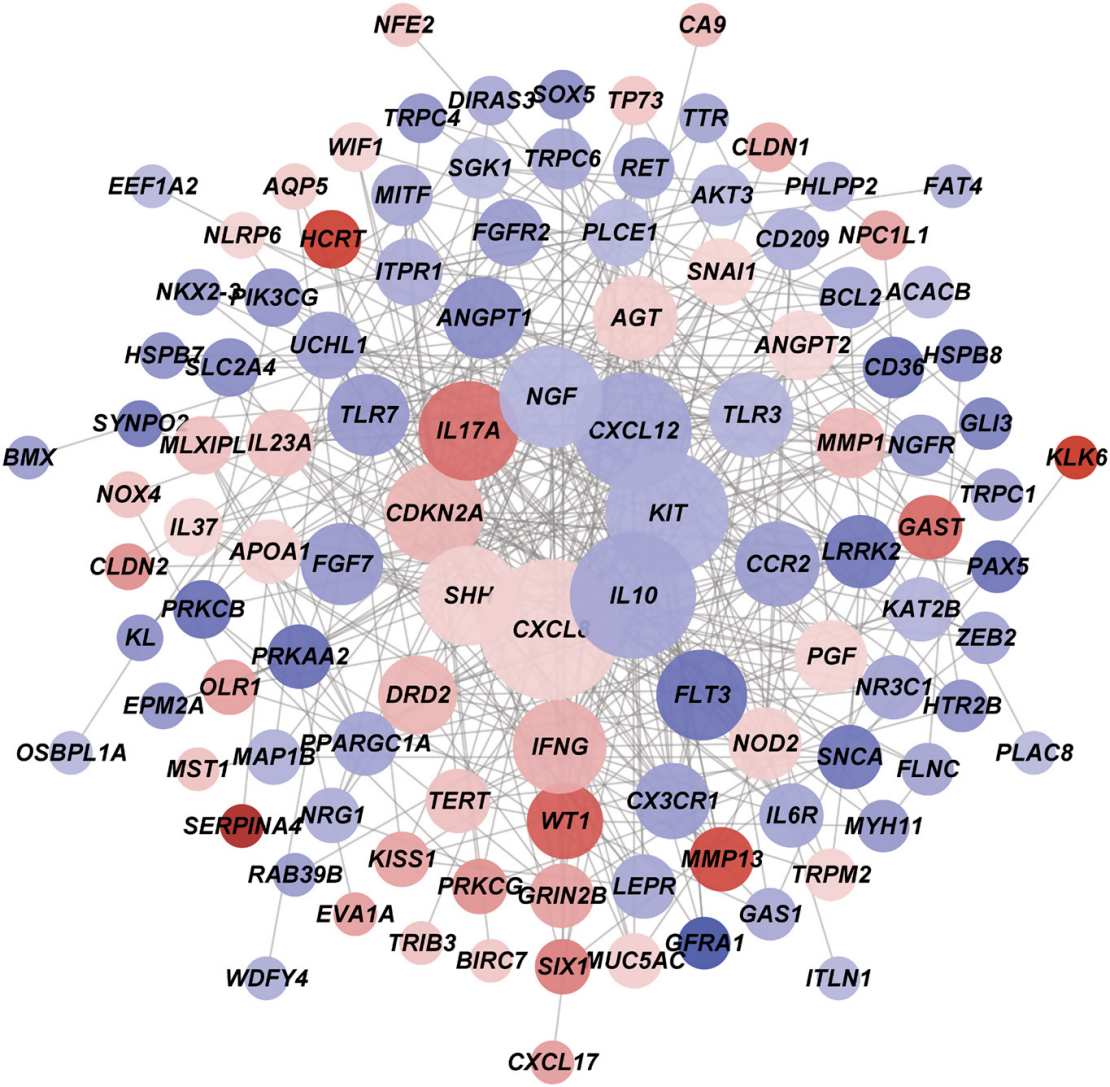
The protein–protein interaction (PPI) network consisting of the autophagy-associated genes. The overall PPI network consisting of the 111 differentially expressed genes (DEGs) associated with autophagy. The node size corresponds to interaction degree, and node color indicates log2[fold change] level. Orange and blue notes upregulation (log2[fold change] >1) and downregulation (log2[fold change] <−1), respectively. The darker the color, the greater the |log_2_(fold change)| value.

A total of 402 interaction pairs between the 129 autophagy-associated DEGs were obtained from the STRING database. Then, the PPI network derived from the interaction pairs was constructed (Figure 3), which consisted of 111 nodes (gene products) and 402 edges (interaction pairs). The 24 nodes with top degrees (≥10) in the PPI network is shown in Table 1, including KIT proto-oncogene, receptor tyrosine kinase (*KIT*, degree = 28), cyclin-dependent kinase inhibitor 2A (*CDKN2A*, degree = 20), and interleukin 17A (*IL17A*, degree = 20).

### Identification of the Prognosis-Associated Genes

**Figure 4.**
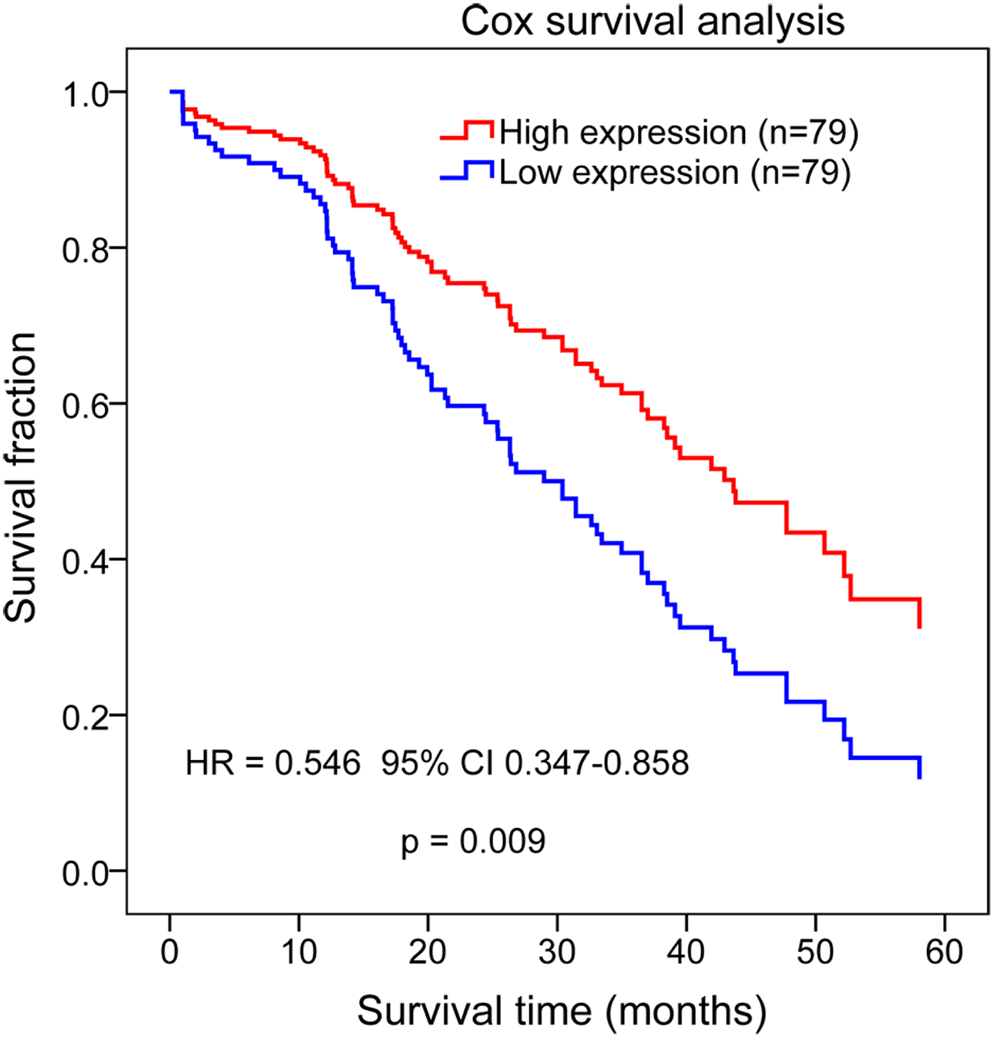
Cox regression analysis showing the association of *PHLPP2* expression with the prognosis of patients with rectum adenocarcinoma. *P* = .009. HR, hazard ratio.

Then, all 111 DEGs including in the PPI network were used to identify the prognostic genes. Univariate Cox regression analysis identified 17 genes were associated with the prognosis of READ (Supplementary Table 2 and Table 2), while multivariate Cox regression analysis showed the *PHLPP2* gene (downregulated) was the only correlated with the survival outcome of READ (HR = 0.442, 95% CI 0.215-0.906, *P* = .026; Supplementary Table 2 and Table 2). Cox regression also indicated that patients with a high expression level of *PHLPP2* had a higher survival ratio (HR = .546, 95% CI 0.347-0.858, *P* = .009; Figure 4).

### Association of PHLPP2 with the Prognosis of Other Human Cancers

**Figure 5.**
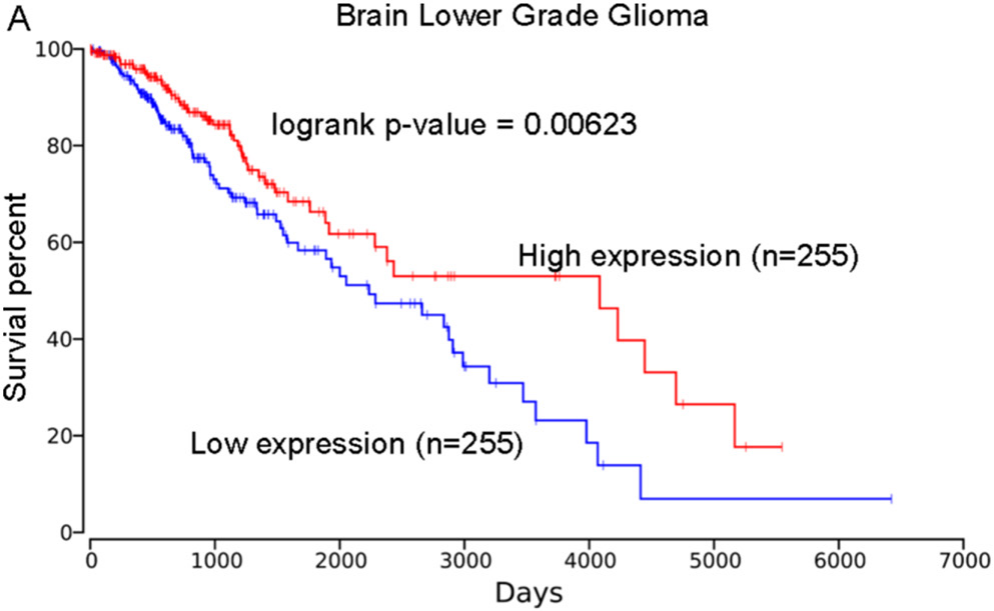
The association of *PHLPP2* expression with the prognosis of brain lower grade glioma. The analysis was performed based on the OncoLnc database, logrank *P* = .00623.

**Figure 6.**
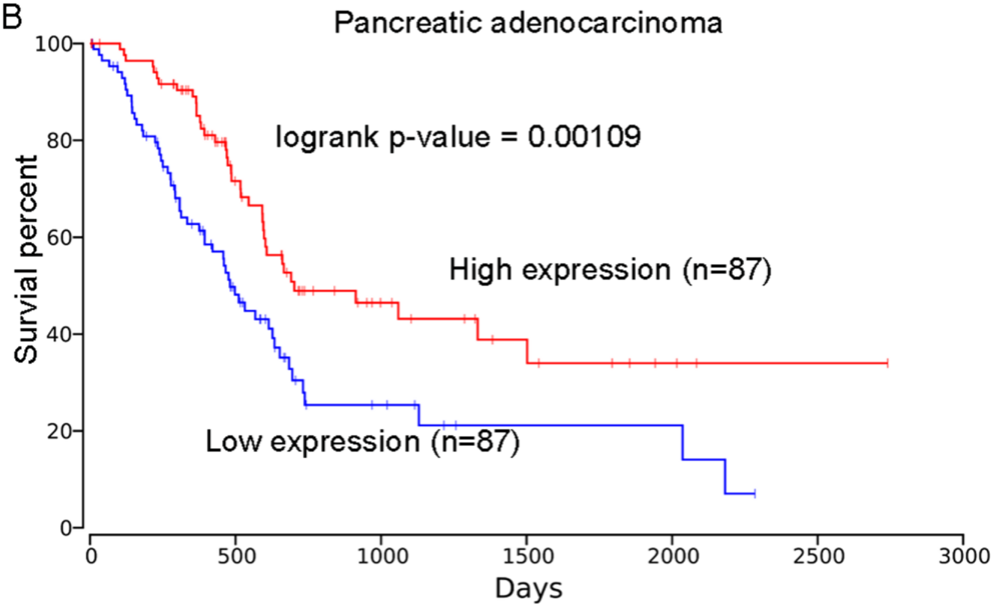
The association of *PHLPP2* expression with the prognosis of pancreatic adenocarcinoma. The analysis was performed based on the OncoLnc database, logrank *P* = .00109.

**Figure 7.**
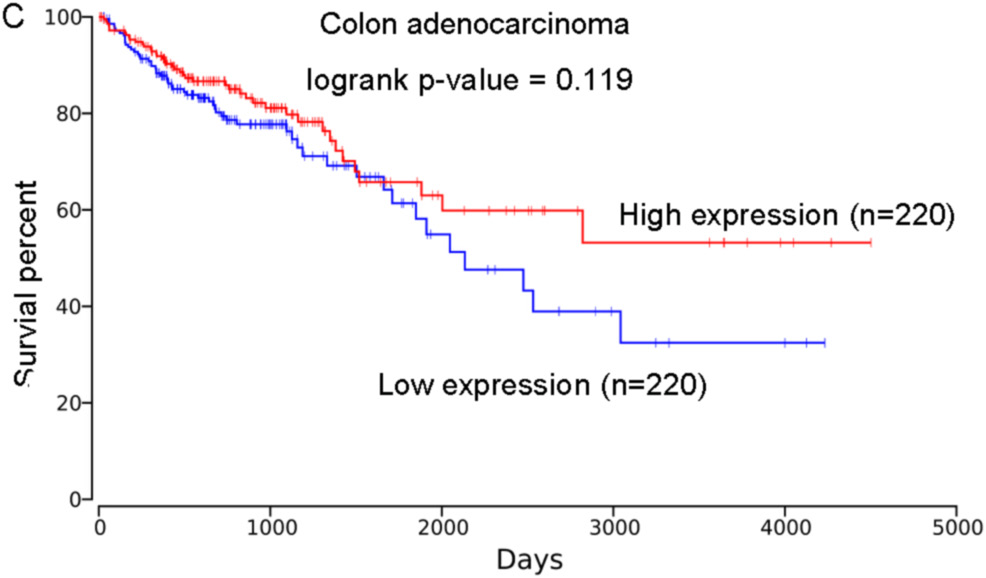
The association of *PHLPP2* expression with the prognosis of colon adenocarcinoma. The analysis was performed based on the OncoLnc database.

Based on the OncoLnc database, we found the high expression of *PHLPP2* was correlated with higher survival percent of patients with brain lower-grade glioma (LGG; logrank *P* = .00623; Figure 5) and pancreatic adenocarcinoma (PAAD; logrank *P* = .00109; Figure 6). These results showed that patients with low expression of *PHLPP2* were at higher risk of poor outcomes for patients with LGG and PAAD. We did not observe its association with the prognosis of other cancers, including the colon adenocarcinoma (COAD, logrank *P* = .119; Figure 7).

### Illustration of the Signaling Pathways Associated with PHLPP2

**Figure 8.**
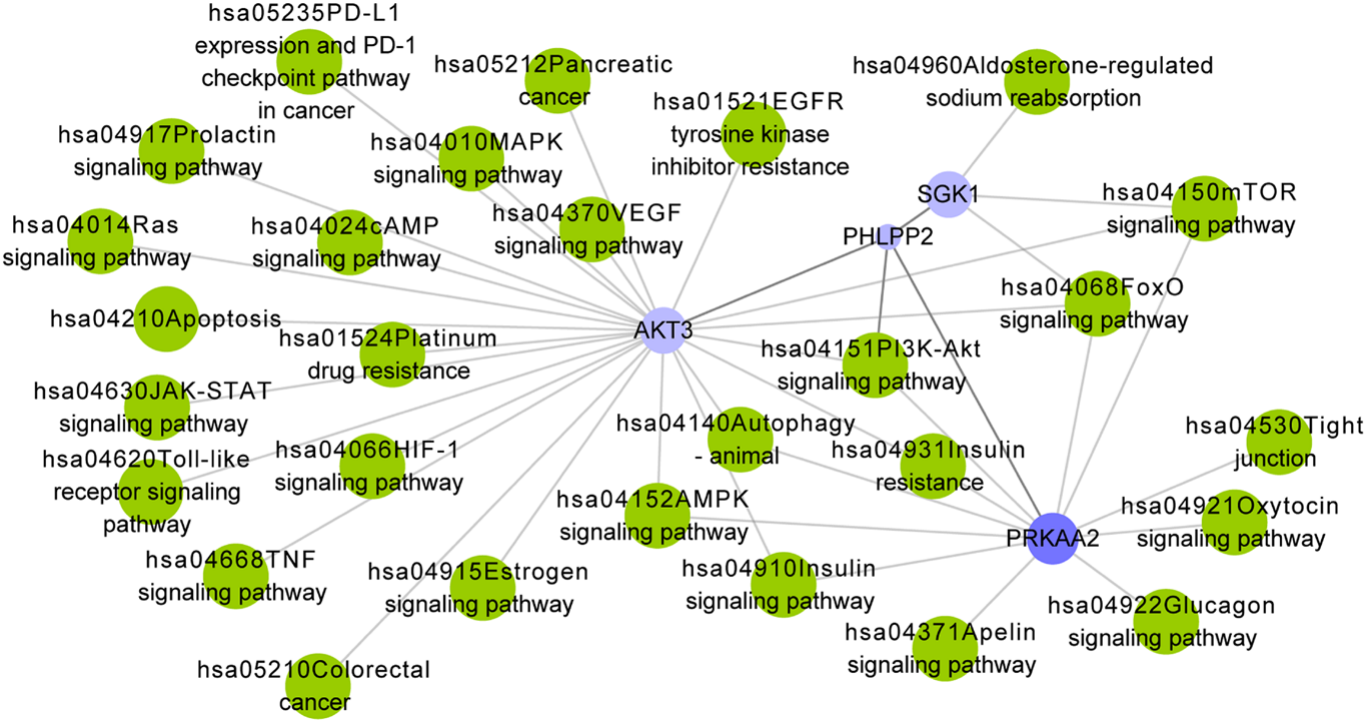
The potential gene-pathway regulatory network involving *PHLPP2* in rectum adenocarcinoma. Blue nodes are downregulated genes (log2[fold change] < −1) in rectum adenocarcinoma. The pathways (green nodes) were extracted from the KEGG database.

To illustrate the potential molecular mechanism mediated by *PHLPP2*, we identified the gene-pathway regulatory network involving *PHLPP2* and the DEGs that interacted with it in the PPI network. *PHLPP2* was directly enriched in the “hsa04151: PI3K-Akt signaling pathway” (Figure 8). The 3 DEGs including downregulated serum/glucocorticoid regulated kinase 1 (*SGK1*), protein kinase AMP-activated catalytic subunit alpha 2 (*PRKAA2*), and AKT serine/threonine kinase 3 (*AKT3*) were associated with 3, 19, and 93 pathways, respectively. However, the gene-pathway network was constructed using the PPI network and the pathways that had been reported to be associated with cancers. Accordingly, 23 and 11 pathways related to *AKT3* and *PRKAA2*, respectively, were retained and used for the construction of the regulatory network. Accordingly, we speculated that the association of *PHLPP2* with the development and prognosis of READ may be associated with various signaling pathways, including “hsa04151: PI3K-Akt signaling pathway,” “hsa04068: FoxO signaling pathway,” “hsa04370: VEGF signaling pathway,” “hsa04066: HIF-1 signaling pathway,” “hsa04630: JAK-STAT signaling pathway,” “hsa04668: TNF signaling pathway,” “hsa04010: MAPK signaling pathway,” and “hsa04210: Apoptosis.”

## Discussion

In this study, the DEGs in the tumor samples were identified and used for the screening of prognosis-associated gene. The results showed that only *PHLPP2* was associated with the prognosis of READ among the known autophagy-associated genes. *PHLPP2* was downregulated in the READ tumor samples as compared with the nontumor adjacent tissues. Also, we found the high expression of *PHLPP2* was associated with a higher survival ratio in patients with READ, LGG, and PAAD but not in COAD. These results might show that *PHLPP2* was a READ-specific prognostic biomarker.

Autophagy is essential for cell survival and differentiation, as well as for homeostasis and disease development. It is a lysosomal degradation pathway that plays a key role in diverse pathologies, including tumorigenesis, neurodegeneration, inflammation, and aging.^22-24^ Accordingly, there has been a tremendous increase in autophagy research in the past 10 years, which has increased the number of autophagy-related genes and proteins reported. Also, targeting autophagy in cancer has been proposed as a future direction.^25-27^ These research studies indicated that autophagy inhibition may be an effective therapeutic strategy in advanced cancers.^25,26,28^ Hence, our present study focused on the association of autophagy-associated genes with the prognosis of READ. Fortunately, we identified that the expression of the *PHLPP2* gene was associated with a high survival probability in patients with READ.

The PHLPP protein directly binds to and inactivates Akt and protein kinase C (PKC).^29,30^ The knockdown of *PHLPP2* is shown to increase the activities of its downstream targets, including *GSK3*, *Akt1*, *Akt3*, and *FoxO*. It inactivates Akt via the dephosphorylation of serine 473.^30,31^ Similarly, PHLPP inactivates PKC via the dephosphorylation of serine 657 (in PCKα).^32^ Accordingly, *PHLPP* expression suppresses cell survival and promotes cell apoptosis in cancer cells.^29,30^ Accordingly, the *PHLPP2* gene is described as a tumor suppressor, as it promotes cell apoptosis and suppresses cell proliferation, invasion, and tumor growth by acting as an antagonist of the PI3K/AKT signaling.^33-37^

The *PHLPP2* gene is expressed in all organs, but at its highest level in the small intestine, followed by the colon, duodenum, testis, and brain.^38^ However, the expression of *PHLPP2* was reported to be lost or greatly decreased in tumor samples.^29,39^ Liu et al^39^ indicated that the expression of *PHLPP1* or *PHLPP2* isoform was lost or decreased in more than 70% of colon tumor specimens as compared with the adjacent normal mucosa. We identified that the PHLPP2 gene was downregulated in the READ tumor samples compared with control, and the downregulation of *PHLPP2* was associated with a poor prognosis. Also, it was only enriched with the PI3K/AKT signaling. These data suggested its crucial role in regulating READ tumor progression. However, the downregulation of the AKT3 gene in READ tumor indicated that the molecular mechanism underlying *PHLPP2*-associated READ progression might not be as simple as they appear, and should be examined carefully.

Given the connection to autophagy, *PHLPP2* did not directly control or regulate autophagy. Peng et al^35^ reported that *PHLPP2* inhibited bladder cancer invasion by promoting the degradation of matrix metalloproteinase 2 (MMP2) via p62-mediated autophagy. Jin et al^40^ identified that *PHLPP2* showed a distinct function in bladder cancer. They found that the inhibition of *PHLPP2* in bladder cancer cells promoted BECN1/Beclin1 degradation, attenuated autophagy, and promoted bladder cancer growth. They also identified that *PHLPP2* mediated the stabilization of BECN1/Beclin1 indirectly by cullin 4A (*CUL4A*) and promoted autophagy.^40^ In other words, the connection between PHLPP2 and autophagy is not direct, and accordingly, the association of PHLPP2 with autophagy in cancers needs to be explored.

## Conclusions

This study showed a positive correlation between *PHLPP2* expression and READ prognosis. The *PHLPP2* gene was downregulated in the tumor samples, and its high expression level was correlated with a higher survival ratio in patients with READ. It may be a READ-specific prognostic biomarker, providing a novel reference for treatment of READ. However, the association of it with PI3K/AKT signaling and the autophagy in READ progression needs to be explored.

## Data Availability

**Data Availability Statement:** The data that support the findings of this study are available from the corresponding author upon reasonable request.

## Figures and Tables

**Table 1.** Top 20 Nodes with Relatively High Interaction Degree in the Protein–Protein Interaction Network

Symbol	logFC	U/down	Degree	Symbol	logFC	U/down	Degree
CXCL8	1.198	Up	34	AGT	1.331	Up	15
IL10	−1.254	Down	29	ANGPT1	−1.626	Down	14
KIT	−1.159	Down	28	FGF7	−1.483	Down	14
CXCL12	−1.282	Down	26	TLR7	−1.476	Down	14
NGF	−1.058	Down	21	DRD2	2.021	Up	13
SHH	1.233	Up	20	CX3CR1	−1.401	Down	12
CDKN2A	1.984	Up	20	WT1	4.563	Up	12
IL17A	3.940	Up	20	LRRK2	−1.912	Down	11
IFNG	2.159	Up	18	PGF	1.166	Up	11
FLT3	−1.955	Down	17	NOD2	1.326	Up	11
CCR2	−1.401	Down	15	ANGPT2	1.082	Up	10
TLR3	−1.078	Down	15	MMP1	1.902	Up	10

FC, fold change.

**Table 2.** Prognosis-Associated Genes in Rectum Adenocarcinoma Patients

Genes	Univariate		Multivariate	
	HR (95% CI)	*P*	HR (95% CI)	*P*
*CA9*	1.155 (1.027-1.299)	.017	1.078 (0.934-1.244)	.330
*CDKN2A*	1.343 (1.092-1.652)	.005	1.204 (0.917-1.582)	.182
*FLNC*	1.228 (1.009-1.493)	.040	1.023 (0.679-1.355)	.462
*GAS1*	1.302 (1.091-1.553)	.003	0.872 (0.505-1.508)	.625
*GLI3*	1.723 (1.062-2.796)	.028	1.476 (0.304-7.178)	.629
*GRIN2B*	0.265 (0.074-0.951)	.042	0.307 (0.078-1.207)	.307
*HSPB7*	1.422 (1.180-1.713)	<.0001	1.398 (0.718-2.722)	.324
*HSPB8*	1.315 (1.070-1.616)	.009	1.074 (0.813-1.353)	.474
*HTR2B*	2.151 (1.299-3.561)	.003	1.464 (0.606-3.537)	.394
*MITF*	1.542 (1.052-2.259)	.026	1.010 (0.242-4.221)	.989
*MYH11*	1.149 (1.014-1.303)	.030	1.194 (0.754-1.892)	.450
*NOX4*	1.713 (1.099-2.670)	.017	1.647 (0.407-6.668)	.484
*NRG1*	0.258 (0.090-0.746)	.012	0.300 (0.081-1.113)	.072
*PGF*	1.429 (1.029-1.984)	.033	1.210 (0.800-1.829)	.367
*PHLPP2*	0.349 (0.191-0.636)	.001	0.442 (0.215-0.906)	**.026**
*PLCE1*	0.529 (0.321-0.872)	.013	0.807 (0.430-1.514)	.505
*SLC2A4*	1.899 (1.218-2.961)	.005	1.537 (0.848-2.785)	.157

*P* < .05 indicates significance. HR, hazard ratio.

**Supplementary Table 1.** The List of the Autophagy-Associated Genes Overlapped between the Differentially Expressed Genes in Rectum Adenocarcinoma

Symbol	logFC	Symbol	logFC	Symbol	logFC	Symbol	logFC
SERPINA4	7.081	TRIB3	1.656	NGF	-1.058	GFRA1	-2.613
KLK6	5.863	IL23A	1.642	TLR3	-1.078	CX3CR1	-1.401
HCRT	5.534	NFE2	1.610	KAT2B	-1.098	TRPC1	-1.408
MMP13	5.151	TP73	1.567	PHLPP2	-1.109	RAB39B	-1.440
WT1	4.563	BIRC7	1.565	MAP1B	-1.131	BMX	-1.445
GAST	4.156	AQP5	1.429	CD209	-1.136	NGFR	-1.446
IL17A	3.940	RDH12	1.407	FAT4	-1.145	NKX2-3	-1.448
SIX1	3.523	AGT	1.331	KIT	-1.159	TLR7	-1.476
ATG9B	3.149	NOD2	1.326	NRG1	-1.167	FGF7	-1.483
CLDN2	3.103	MUC5AC	1.312	WDFY4	-1.171	MYH11	-1.499
PRKCG	2.985	MACC1	1.284	ITPR1	-1.200	SRPX	-1.513
MAPK15	2.752	WIF1	1.244	HPGD	-1.207	EPM2A	-1.540
CXCL17	2.670	TRPM2	1.238	AKR1B10	-1.232	PIK3CG	-1.554
EVA1A	2.638	NLRP6	1.234	DIRAS3	-1.238	TRPC4	-1.570
OLR1	2.638	APOA1	1.234	MITF	-1.252	HSPB7	-1.572
GRIN2B	2.582	SHH	1.233	IL10	-1.254	KL	-1.586
NPC1L1	2.527	CXCL8	1.198	GAS1	-1.261	HTR2B	-1.598
KISS1	2.521	ABCB6	1.194	TRPC6	-1.270	SOX5	-1.622
CLDN1	2.334	IL37	1.177	LEPR	-1.278	ANGPT1	-1.626
FUT1	2.168	PGF	1.166	CXCL12	-1.282	SLC2A4	-1.645
IFNG	2.159	SNAI1	1.134	BCL2	-1.285	HSPB8	-1.696
DRD2	2.021	FBXO2	1.112	TTR	-1.296	GLI3	-1.781
CDKN2A	1.984	ANGPT2	1.082	RET	-1.307	CD36	-1.900
GJB5	1.973	ACACB	-1.013	ZEB2	-1.310	SNCA	-1.911
CA9	1.949	PLCE1	-1.019	PPARGC1A	-1.311	SYNPO2	-1.911
MMP1	1.902	AKT3	-1.023	NR3C1	-1.328	LRRK2	-1.912
KIF25	1.822	PLAC8	-1.024	FLNC	-1.334	FLT3	-1.955
MLXIPL	1.724	EEF1A2	-1.040	NPR3	-1.340	PAX5	-1.958
TERT	1.724	SGK1	-1.041	FAM110B	-1.365	PRKCB	-2.019
NOX4	1.704	OSBPL1A	-1.042	UCHL1	-1.391	RNF152	-2.047
MST1	1.693	ITLN1	-1.053	CCR2	-1.401	PRKAA2	-2.050
				PLA2G5	-2.050	TINCR	-2.129

FC, fold change.

**Supplementary Table 2.** Cox Regression Analysis for the Prognosis-Associated Genes in Rectum Adenocarcinoma Patients from the TCGA Database

Genes	Univariate		Multivariate
	HR (95% CI)	*P*	HR (95% CI)
*P*
	ACACB	1.206 (0.673-2.160)	.529		
AGT	0.967 (0.807-1.159)	.717		
AKT3	1.383 (0.937-2.040)	.103		
ANGPT1	0.860 (0.445-1.664)	.655		
ANGPT2	0.960 (0.601-1.533)	.864		
APOA1	0.891 (0.698-1.137)	.353		
AQP5	0.720 (0.324-1.598)	.419		
BCL2	0.680 (0.414-1.117)	.128		
BIRC7	0.885 (0.639-1.226)	.461		
BMX	0.376 (0.177-0.795)	.461		
CA9	1.155 (1.027-1.299)	**.017**	1.078 (0.934-1.244)	.3307
CCR2	1.394 (0.810-2.398)	.231		
CD209	1.153 (0.828-1.606)	.399		
CD36	1.323 (0.898-1.949)	.156		
CDKN2A	1.343 (1.092-1.652)	**.005**	1.204 (0.917-1.582)	.182
CLDN1	0.854 (0.704-1.037)	.111		
CLDN2	1.021 (0.911-1.144)	.724		
CX3CR1	1.247 (0.618-2.513)	.538		
CXCL12	1.090 (0.844-1.409)	.507		
CXCL17	1.096 (0.858-1.400)	.461		
CXCL8	1.004 (0.886-1.136)	.955		
DIRAS3	1.101 (0.726-1.670)	.651		
DRD2	1.068 (0.860-1.326)	.551		
EEF1A2	1.135 (0.939-1.371)	.191		
EPM2A	0.985 (0.296-3.273)	.980		
EVA1A	0.891 (0.706-1.128)	.341		
FAT4	0.795 (0.445-1.422)	.439		
FGF7	1.144 (0.841-1.555)	.392		
FGFR2	1.218 (0.912-1.627)	.182		
FLNC	1.228 (1.009-1.493)	**.040**	1.023 (0.679-1.355)	.462
FLT3	0.478 (0.074-3.072)	.437		
GAS1	1.302 (1.091-1.553)	**.003**	0.872 (0.505-1.508)	.625
GAST	1.405 (0.913-2.162)	.122		
GFRA1	1.198 (0.681-2.109)	.530		
GLI3	1.723 (1.062-2.796)	**.028**	1.476 (0.304-7.178)	.629
GRIN2B	0.265 (0.074-0.951)	**.042**	0.307 (0.078-1.207)	.307
HCRT	1.348 (0.832-2.183)	.225		
HSPB7	1.422 (1.180-1.713)	**<.0001**	1.398 (0.718-2.722)	.324
HSPB8	1.315 (1.070-1.616)	**.009**	1.074 (0.813-1.353)	.474
HTR2B	2.151 (1.299-3.561)	**.003**	1.464 (0.606-3.537)	.394
IFNG	1.050 (0.454-2.429)	.909		
IL10	1.823 (0.865-3.842)	.114		
IL17A	0.970 (0.511-1.840)	.926		
IL23A	1.151 (0.871-1.523)	.323		
IL37	0.921 (0.673-1.261)	.609		
IL6R	0.827 (0.563-1.214)	.332		
ITLN1	0.979 (0.906-1.058)	.598		
ITPR1	1.196 (0.688-2.076)	.526		
KAT2B	0.782 (0.500-1.222)	.280		
KISS1	1.104 (0.810-1.507)	.530		
KIT	0.927 (0.665-1.291)	.652		
KL	1.236 (0.700-2.184)	.465		
KLK6	1.025 (0.915-1.1150)	.668		
LEPR	0.851 (0.474-1.528)	.589		
LRRK2	1.073 (0.502-2.293)	.855		
MAP1B	1.224 (0.867-1.729)	.251		
MITF	1.542 (1.052-2.259)	**.026**	1.010 (0.242-4.221)	.989
MLXIPL	0.903 (0.738-1.106)	.325		
MMP1	0.955 (0.854-1.068)	.420		
MMP13	1.124 (0.966-1.597)	.091		
MST1	0.767 (0.534-1.102)	.151		
MUC5AC	1.023 (0.821-1.273)	.841		
MYH11	1.149 (1.014-1.303)	**.030**	1.194 (0.754-1.892)	.450
NFE2	1.118 (0.807-1.550)	.502		
NGF	1.523 (0.980-2.366)	.061		
NGFR	1.256 (0.982-1.608)	.070		
NKX2-3	1.149 (0.795-1.660)	.461		
NLRP6	1.089 (0.864-1.373)	.498		
NOD2	1.041 (0.675-1.605)	.857		
NOX4	1.713 (1.099-2.670)	**.017**	1.647 (0.407-6.668)	.484
NPC1L1	1.027 (0.8814-1.295)	.825		
NR3C1	1.149 (0.781-1.690)	.480		
NRG1	0.258 (0.090-0.746)	**.012**	0.300 (0.081-1.113)	.072
OLR1	1.259 (0.993-1.597)	.057		
OSBPL1A	0.962 (0.668-1.384)	.834		
PAX5	0.984 (0.616-1.574)	.948		
PGF	1.429 (1.029-1.984)	**.033**	1.210 (0.800-1.829)	.367
PHLPP2	0.349 (0.191-0.636)	**.001**	**0.442 (0.215-0.906)**	**.026**
PIK3CG	1.060 (0.552-2.035)	.860		
PLAC8	1.000 (0.858-1.166)	.999		
PLCE1	0.529 (0.321-0.872)	**.013**	0.807 (0.430-1.514)	.505
PPARGC1A	0.724 (0.491-1.069)	.104		
PRKAA2	0.598 (0.334-1.069)	.083		
PRKCB	1.161 (0.659-2.046)	.606		
PRKCG	0.982 (0.727-1.327)	.909		
RAB39B	0.733 (0.211-2.542)	.624		
RET	1.103 (0.682-1.782)	.690		
SERPINA4	0.975 (0.745-1.275)	.852		
SGK1	0.812 (0.583-1.130)	.216		
SHH	0.949 (0.718-1.255)	.715		
SIX1	1.443 (0.851-2.449)	.174		
SLC2A4	1.899 (1.218-2.961)	**.005**	1.537 (0.848-2.785)	.157
SNAI1	1.092 (0.780-1.530)	.608		
SNCA	0.957 (0.569-1.610)	.869		
SOX5	0.738 (0.126-4.310)	.736		
SYNPO2	1.212 (0.995-1.475)	.056		
TERT	1.281 (0.750-2.188)	.365		
TLR3	0.693 (0.426-1.128)	.141		
TLR7	1.456 (0.801-2.647)	.217		
TP73	1.086 (0.637-1.853)	.762		
TRIB3	0.816 (0.648-1.027)	.083		
TRPC1	1.575 (0.822-3.018)	.171		
TRPC4	1.079 (0.238-4.893)	.922		
TRPC6	1.210 (0.452-3.326)	.704		
TRPM2	1.127 (0.887-1.432)	.327		
TTR	0.990 (0.805-1.217)	.923		
UCHL1	1.106 (0.901-1.359)	.336		
WDFY4	1.130 (0.572-2.234)	.725		
WIF1	0.924 (0.772-1.105)	.386		
WT1	0.955 (0.680-1.343)	.793		
ZEB2	1.266 (0.809-1.980)	.302		

HR, hazard ratio. CI, confidential interval. TCGA, The Cancer Genome Atlas.
